# Outcomes of colorectal cancer surgery in the nonagenarians: 20-year result from a tertiary center

**DOI:** 10.1186/s12893-019-0623-4

**Published:** 2019-10-28

**Authors:** Toi Yin Chan, Chi Chung Foo, Wai Lun Law, Oswens Lo

**Affiliations:** 1 Department of Surgery, Queen Mary Hospital, Hong Kong, China; 2 Department of Surgery, The University of Hong Kong, 102 Pokfulam Road, Hong Kong, China

**Keywords:** Nonagenarian, Colorectal cancer, Elderly

## Abstract

**Background:**

There is a foreseeable trend that life expectancy is on the rise in many parts of the world. More and more patients will present with colorectal cancer at extreme old age and advanced age is a well-known risk factor for adverse outcomes after surgery. The aim of this study is to evaluate the outcomes of colorectal cancer surgery in patients aged 90 or above.

**Method:**

A retrospective analysis of consecutive patients aged 90 or above who underwent operations for colorectal cancer between January 1996 and December 2015 was performed. The primary outcomes were the complications rate, 30-day and 180-day mortality rates.

**Results:**

A total of 57 patients were included in the analysis. The majority of them were women (64.9%). The median age was 92 years. Most of the surgery was of curative intent (77.2%), performed under elective setting (57.9%) and with open approach (78.9%). 36.8% of patients had postoperative complications, with pneumonia being the commonest. The 30-day and 180-day mortality rate was 7 and 31.6% respectively. History of ischemic heart disease and surgery under emergency setting were predictors of postoperative complications. Pneumonia, preoperative leukocytosis and Charlson comorbidity score ≥ 9 were predictors of 180-day mortality. The one and two-year survival rate for elective surgery was 69.7 and 54.5% respectively.

**Conclusion:**

The outcomes of colorectal cancer surgery for nonagenarians could be favorable in a selected group of patients. Future study on better risk profiling and ways to improve outcomes is warranted.

## Background

Life expectancy is rising worldwide, and according to statistics from the United Nations in 2015, 0.8% of the populations in developed countries were aged 90 or above [1]. This number is expected to triple by 2065, increasing to 3.3%. Citizens of Hong Kong are known to enjoy some of the longest life-expectancies in the world, reaching 81.32 and 87.34 years for men and women respectively [2]. According to the latest projection from the Hong Kong Census and Statistics Department, by year 2066, 428,700 citizens in Hong Kong will be of 90 years old or above, constituting 5.6% of the entire population [3]. With improving socio-economic environments and the availability of high quality health care, a similar trend was also observed in other developed regions, such as Australia and some parts of Europe [4]. As a result of this increase in life expectancy, it is increasingly common for physicians to treat patients at the extremes of age. Elderly patients are more likely to have lower physiological reserve and higher American Society of Anesthesiologists (ASA) scores [5].

Colorectal cancer is the leading malignancy in many countries and is especially common in elderly individuals [6]. The decision whether to operate on this group of patients is often difficult due to the higher risk of complications and mortality [7]. To date, there are limited data on the outcome of colorectal cancer surgery in the extreme elderly.

This study aimed to evaluate the outcomes of colorectal cancer surgery for nonagenarian patients, i.e. patients who were between 90 and 99 years old at the time of surgery, in a tertiary referral center over a 20-year period.

## Methods

This was a retrospective observational study approved by the Institutional Review Board of the University of Hong Kong/Hospital Authority (UW 18–229). Between January 1996 and December 2015, all patients who suffered from histologically proven adenocarcinoma of the colon and rectum and underwent surgery were entered into a prospectively maintained database. Patients who were lost to follow-up or had incomplete data were excluded. Patients who were aged 90 or above at the time of surgery were reviewed. All patients received broad spectrum antibiotics on induction, covering common colonic microbes such as gram negative bacilli and anaerobes. Bowel preparation was administered to patients undergoing elective surgery unless the tumor was partially obstructing and as a result patients were unable to tolerate the iso-osmotic bowel cleansing agent. The following baseline demographics were retrieved: ASA score, Eastern Cooperative Oncology Group (ECOG) performance status [8], Charlson comorbidity index (CCI) [9], preoperative biochemical markers (serum creatinine, white cell count, albumin, hemoglobin levels), acuity and approach of surgery, tumor characteristics, treatment types, morbidities, 30-day mortality, 180-day mortality, and survival rates. The primary endpoints included postoperative complications, and 30-day and 180-day mortality rates. The secondary endpoints were the factors that could predict complications and mortality.

Univariate analysis was performed on various clinical parameters. Categorical variables were analyzed using the Fisher exact test and the χ^2^ test where appropriate. Continuous variables were analyzed using the independent sample t-test. All comparisons were two-sided, and a *p* value of < 0.05 was considered statistically significant. Multivariate analysis was performed to identify the predictors for overall complications, and 30-day and 180-day mortality, using a forward stepwise model. Variables with significant univariate association, defined by a *p* value of < 0.1, were included in the multivariate analysis. The survival rates of the elective and emergency surgery groups were estimated with the Kaplan-Meier curve. Statistical analysis was performed by SPSS version 20 (IBM Corporation, Armonk, NY, USA).

## Results

During the study period, there were a total of 68 patients who were aged 90 or above at the time of surgery. After excluding those with incomplete data, 57 patients were included in the analysis; the majority of them (64.9%) were female. The age of patients ranged from 90 to 101 years, with a median age of 92 years; 42.1, 52.6, and 5.3% of the patients were ASA 2, 3, and 4, respectively (Table 1), and 36.8, 49.1, and 14.0% of the patients were ECOG 2, 3, and 4, respectively. The CCI ranged from 6 to 13, with a median of 8; 73.7% of patients were non-smokers, and 56.1% had hypertension, while 10.5% had a history of ischemic heart disease. The majority of the operations (77.2%) were of curative intent; 57.9% were performed under the elective setting and 21.1% with the laparoscopic approach. The most commonly performed operation was right hemicolectomy (29.8%). The length of hospital stay ranged from 3 to 57 days, with a median of 9 days. The median follow-up period was 15.9 months (range, 0.3–115.8 months). None of the patients were lost to follow-up before death.

**Table 1:** Patient demographics, tumor characteristics and surgical procedures

	n	%
Sex		
Male	20	35.1
Female	37	64.9
ASA		
2	24	42.1
3	30	52.6
4	3	5.3
ECOG		
2	21	36.8
3	28	49.1
4	8	14.0
Tumor location		
Colon	38	66.7
Rectum	19	33.3
Stage^		
1	4	7.0
2	23	40.4
3	20	35.1
4	6	10.5
Unknown	4	7.0
Procedures		
Right / extended right hemicolectomy / transverse colectomy	25	43.9
Hartmann’s procedure	9	15.8
Left hemicolectomy / sigmoidectomy / anterior resections	8	14.0
Low anterior resection	7	12.3
Subtotal colectomy	3	5.3
Diversion stoma	3	5.3
Bypass	2	3.5

^According to the 8th American Joint Committee on Cancer (AJCC) staging classification

A total of 21 patients (36.8%) developed postoperative complications, the majority of which was pneumonia (14.0%). Four patients developed respiratory failure requiring tracheostomy after surgery. Four patients (7.0%) developed paralytic ileus, and all resolved with conservative treatment. Five patients (8.8%) suffered from myocardial infarction or arrhythmia. Two patients (3.5%) suffered from urinary tract infections and were managed with antibiotics. One (1.8%) suffered from stroke and was treated with aspirin. Two patients (3.5%) underwent re-operations within the same admission. One of them had anastomotic leakage and intra-abdominal collection after an elective palliative bypass for a cecal tumor. The other developed recurrent inguinal hernia with strangulation after an emergency right hemicolectomy and synchronous bilateral inguinal herniorrhaphy for cancer of the transverse colon; 86% of the patients were discharged home after various period of rehabilitation. The overall complication rates were 24.2 and 50.0% for elective and emergency operations, respectively.

Four patients died within the first 30 postoperative days, and three were operated under the emergency setting. The 30-day mortality rate was 3% (1/33) for elective surgery and 12.5% (3/24) for emergency surgery. The overall 30-day mortality rate was 7%, with postoperative pneumonia being the cause of death in all (Table 2).

**Table 2:** Causes of death at various time intervals after operation

Days post-operation	%
Within 30 days	
Pneumonia	100
Within 180 days	
Pneumonia	42.9
Terminal malignancy	21.4
Unknown	14.3
Sepsis	7.1
Stroke	7.1
Dermatomyositis	7.1
Beyond 180 days	
Pneumonia	51.5
Terminal malignancy	21.2
Unknown	12.1
Stroke	6.1
Acute myocardial infarction	3.0
Perforated viscus	3.0
Cholangitis	3.0

The overall 180-day mortality rate was 31.6%. The 6-month survival rate was 75.8% (25/33) for elective surgery and 58.3% (14/24) for emergency surgery. The most common cause of death was pneumonia (42.9%), followed by terminal malignancy (21.4%). There were only three more deaths from 6 months to 1 year after surgery, and they were all attributed to terminal malignancy.

**Fig. 1:**
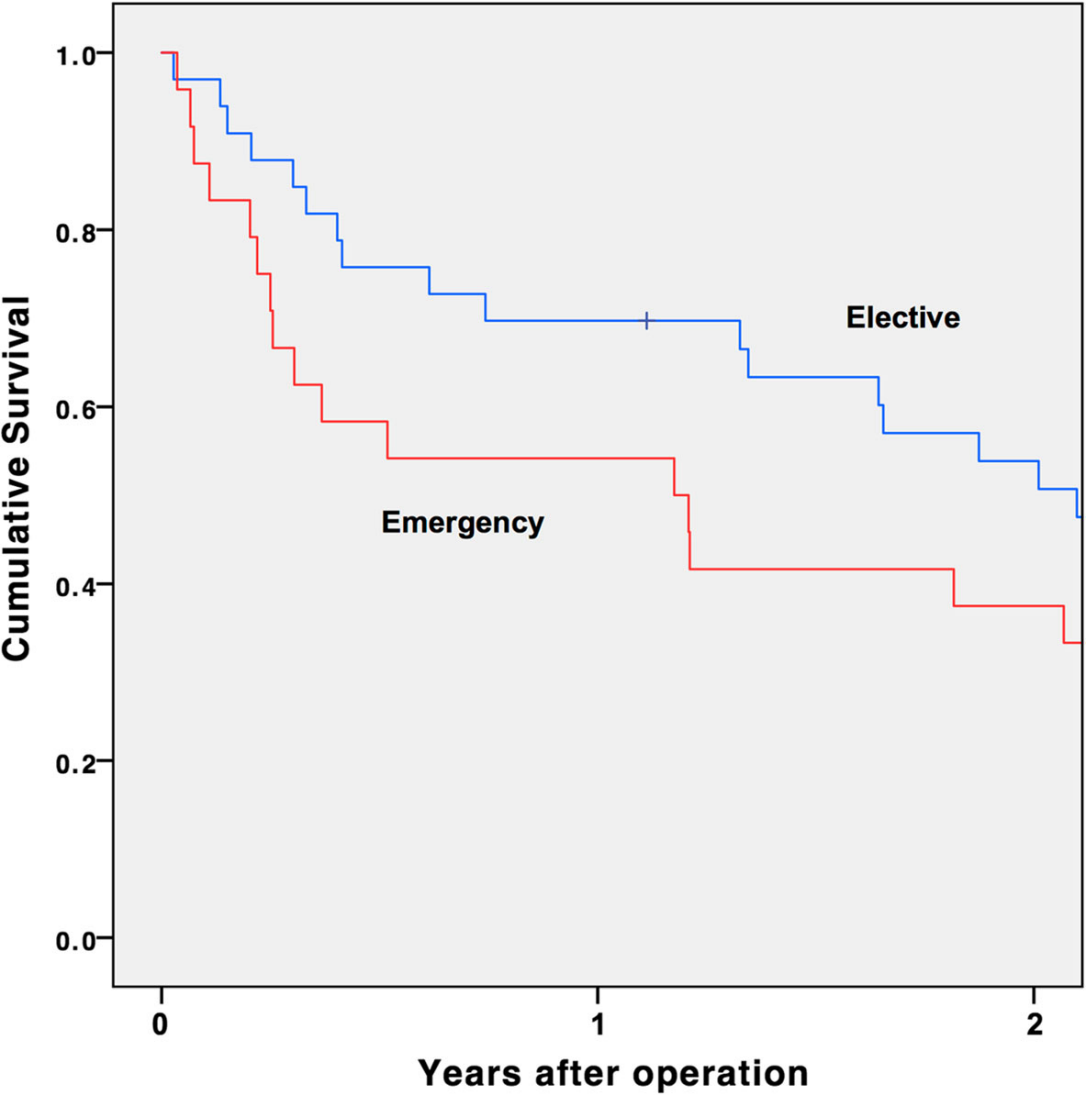
Kaplan-Meier curves showing the cumulative survival after elective and emergency surgery

The 1 and 2-year survival rates for elective surgery were 69.7% (23/33) and 54.5% (18/33), respectively. The 1 and 2-year survival rates for emergency surgery were 54.2% (13/24) and 37.5% (9/24), respectively (Fig. 1).

The factors associated with postoperative complications are shown in Table 3. Patients with ASA 4 (*p* = 0.039), history of ischemic heart disease (*p* = 0.017), colon cancer (*p* = 0.031), and those who underwent emergency surgery (*p* = 0.044) were at significantly higher risk of developing complications. ASA 4 (*p* = 0.002), history of ischemic heart disease (*p* = 0.031), and emergency surgery (*p* = 0.059) were also associated with postoperative respiratory complications (data not shown).

**Table 3:** Factors associated with postoperative complications, 30-day mortality & 180-day mortality

Factors	Postoperative complications		30-day mortality		180-day mortality	
	(%)	*p*	(%)	*p*	(%)	*p*
Sex		0.568		0.119		0.683
Male	40		15		35	
Female	32.4		2.7		29.7	
ASA		0.039		0.011		0.028
II-III	31.5		3.7		27.8	
IV	100		66.7		100	
ECOG		1.0		0.464		0.094
< 4	34.7		6.1		26.5	
≥ 4	37.5		12.5		62.5	
CCI		0.53		0.042		0.025
< 9	32.6		2.3		23.3	
≥ 9	42.9		21.4		57.1	
Chronic smoker		1.0		1.0		1.0
No	34.5		7.3		32.7	
Yes	50		0		0	
Hypertension		0.898		0.623		0.526
No	36		4		36	
Yes	34.3		9.3		28.1	
Diabetes mellitus		1.0		0.464		0.698
No	34.7		6.1		30.6	
Yes	37.5		12.5		37.5	
Ischemic heart disease		0.017		0.051		0.072
No	29.4		3.9		27.5	
Yes	83.3		33.3		66.7	
Cerebrovascular disease		1.0		0.464		1.0
No	34.7		6.1		32.7	
Yes	37.5		12.5		25	
Respiratory disease		0.687		0.07		1.0
No	34		4		32	
Yes	42.8		28.6		28.6	
Chronic renal failure		1.0		1.0		1.0
No	35.8		7.5		32.1	
Yes	25		0		25	
Cirrhosis		1.0		1.0		1.0
No	35.7		7.1		32.1	
Yes	0		0		0	
Current antiplatelet use		0.332		0.315		0.646
No	32.7		5.8		30.8	
Yes	60		20		40	
Surgical urgency		0.044		0.3		0.162
Elective	24.2		3		24.2	
Emergency	50		12.5		41.7	
Surgical approach		0.182		0.569		0.303
Laparoscopic	16.7		0		16.7	
Open	40		8.9		35.6	
Surgical intent		0.346		1.0		0.087
Curative	38.6		6.8		25	
Palliative	23.1		7.7		53.8	
Tumor location		0.031		1.0		0.227
Colon	44.7		7.9		36.8	
Rectum	15.8		5.2		21.1	
Disease staging		0.651		1.0		0.351
1–3	36.2		6.4		27.7	
4	16.7		0		50	
Serum biochemistry^						
Hemoglobin		0.173		0.852		0.189
White cell count		0.566		0.981		0.021
Albumin		0.629		0.041		0.008
Creatinine		0.675		0.473		0.885
Overall complications				0.012		0.005
No			0		18.9	
Yes			20		55	
Pulmonary complications				< 0.001		0.001
No			0		22.4	
Yes			50		87.5	
Reoperation				1.0		0.536
No			7.3		30.9	
Yes			0		50	

^Analysis done by independent sample T test

ASA 4 (*p* = 0.011), CCI score ≥ 9 (*p* = 0.042), serum albumin level (*p* = 0.041), pneumonia (*p* ≤ 0.001), and overall complications (*p* = 0.012) had a significant association with 30-day mortality. All of these, together with preoperative leukocytosis (*p* = 0.021) were also associated with 180-day mortality.

Multivariate analysis revealed that emergency surgery (*p* = 0.025, odds ratio 4.2) and a history of ischemic heart disease (*p* = 0.016, odds ratio 17.6) were predictors of postoperative complications. Pneumonia (*p* = 0.004, odds ratio 33.1), preoperative leukocytosis (*p* = 0.005, odds ratio 1.3), and CCI score of ≥9 (*p* = 0.059, odds ratio 4.8) were independent predictors of 180-day mortality (Table 4).

**Table 4:** Multivariate analysis to identify the predictors of overall complications, 30-day mortality and 180-day mortality

	*p*	Odds Ratio	95% CI
Overall complications			
ASA 4	0.105		
Ischemic heart disease	0.016	17.6	1.7–184.0
Emergency surgery	0.025	4.2	1.2–14.8
Tumor location	0.208		
30-day mortality			
ASA 4	0.465		
CCI (< 9 / ≥9)	0.157		
Ischemic heart disease	0.465		
Respiratory disease	0.102		
Overall complications	1.000		
Pulmonary complications	0.997		
Serum albumin level	0.199		
180-day mortality			
ASA 4	0.406		
ECOG (< 4 / ≥4)	0.113		
CCI (< 9 / ≥9)	0.059	4.8	0.9–24.1
Ischemic heart disease	0.235		
Surgical intent	0.201		
Overall complications	0.080		
Pulmonary complications	0.004	33.1	3.1–358.5
Serum albumin level	0.195		
White cell count	0.005	1.3	1.1–1.6

There were 10 patients operated on during the last 5 years of the study period. This group of patients outperformed their predecessors by achieving a better overall 2-year survival (Table 5).

**Table 5:** A comparison of patients enrolled in the first 15 years and the last 5 years of the study period

	First 15 years	Last 5 years	*p* value
Complications	38.3%	40%	0.271
30-day mortality	8.5%	10%	0.339
180-day mortality	34%	20%	0.386
1-year survival	59.6%	70%	0.224
2-year survival	40.4%	60%	0.023

## Discussion

According to the Global Health and Ageing report from the World Health Organization, the number of people aged 65 or above was projected to increase from an estimated 524 million in 2010 to nearly 1.5 billion in 2050, with much of these in developing countries. The recent increase in life expectancy and improvement in health care was associated with a change in the leading cause of death from infectious to chronic diseases, including cancer.

There is ample evidence that major surgery could be safely performed in the elderly [10], typically in the age group of 70 or above. However, there are still concerns about performing surgery in very old patients. In previous studies, the mortality rate after surgery for colorectal malignancies ranged from 3.4 to 7% [11, 12]. Data on the operative outcomes after colorectal resections for nonagenarians were scarce. Indeed, age and comorbidities were shown to have a negative impact on the surgical outcomes. A Dutch study showed that advanced age and comorbidities were associated with early postoperative mortality after surgery for gastrointestinal malignancies [13]. Furthermore, Yeo et al. demonstrated that the incidence of adverse events and the likelihood of postoperative mortality increased when patients were older than 75, and the risk increased further if the age was greater than 85 [14]. However, with an ageing population that continues to grow, surgeons will increasingly find themselves managing patients in this age group; thus, there is a requirement to evaluate whether or not they are good candidates for surgery.

One argument that supported elective surgical interventions in this group of patients was that they would present with surgical emergencies, such as intestinal obstruction, tumor perforation, and bleeding, and were subjected to an even higher surgical risk [15, 16]. Owing to the lack of health awareness, social neglect, and poor accessibility to healthcare service, elderly patients are already more likely to present late with advanced disease. Worse still, they often present with an acute surgical condition that requires emergency surgery [7]. A United States population-wide study showed that the percentage of cases operated on an emergency basis rose from 5.9%, for those < 65 years of age, to 8.9% for those older than 85 years of age [14].

This study demonstrated that, in selected patients, acceptable outcomes could be achieved in nonagenarian patients. The postoperative mortality in this series was comparable to that previously reported in the literature [17]. The 30-day mortality rate after elective resections in the nonagenarian patients was comparable to that of the younger elderly population. For example, statistics from a population-based study from the American College of Surgeons National Surgical Quality Improvement Program database showed that the 30 day mortality after colon and rectal surgery was 4.1 and 2.4%, respectively for patients between 75 and 84 years of age [14]. It was also shown that more than half of the patients in this study cohort survived more than 2 years after elective surgery. Logically, the longer-term survival, e.g. 5 years, could not be compared to the younger elderly patients.

One important parameter of surgical outcome was the 6 month survival rate. As shown in this study, a substantial portion of the patients succumbed during the first 6 months, despite surviving the first 30 days. A previous study has shown that a major portion of elderly postoperative mortality occurred after the index hospitalization [14]. Furthermore, many were discharged to nursing homes or institutional care facilities after the index operation. This highlighted the need for improved support for these individuals in the early post-hospitalization period.

Operation risk evaluation and patient selection is of paramount importance in treating nonagenarian patients, and various methods have been proposed to quantify a patient’s health status and to predict surgical outcomes [18]. Elderly individuals who are functionally dependent and malnourished are more prone to morbidities and mortality. The Comprehensive Geriatric Assessment takes into account the physical health, functional status, psychological health, and social support of this group of patients [19]. In addition, the Portsmouth-Physiological and Operative Severity Score for the enumeration of Mortality and morbidity score is another popular tool widely adopted in the United Kingdom, and studies have shown that it can reliably predict mortality after emergency laparotomy in elderly patients [20]. Recently there has been increasing interest in using sacropenia and frailty to predict surgical outcome after operations for gastrointestinal malignancies [21, 22].

In this study, three scoring systems that are well-validated and relatively easy to calculate were used: ASA, CCI, and ECOG performance status [23]. The ECOG is one of the most commonly used research tools to quantify the performance status of cancer patients [8]. The CCI was first proposed by Mary Charlson in 1987 and is the most extensively studied comorbidity index [9, 24]. It focuses on a list of selected co-morbidities, while the ECOG assesses the functional performance; they have both been shown to predict survival independent of the treatments [18]. This study confirmed that ASA 4 was associated with postoperative morbidity and mortality and that patients with a CCI score of ≥9 were less likely to survive beyond 180 days after surgery. Performance status, as reflected by the ECOG score, was not a predictor of postoperative outcome in this study.

This study also showed that hypoalbuminemia was associated with a higher post-operative mortality rate. A previous study had established a similar relationship between hypoalbuminemia and a higher incidence of postoperative complications [25]. Furthermore, the serum albumin level reflects the nutritional status, and elderly patients with underlying malignant condition are at risk of malnutrition.

Rigberg et al. reviewed their experience with major general surgical operations in nonagenarians and reported an overall morbidity rate of 57%, with pneumonia being the most common postoperative complication [26]. This was in line with the current study, which identified pneumonia with respiratory failure as the most common postoperative complication and the leading cause of mortality, despite having a high proportion of non-smokers in the current study. A meta-analysis showed that laparoscopic colorectal resection has a lower incidence of postoperative pneumonia compared to the open approach, with an odds ratio of 0.53 [27].

An Australian study by Yap et al. on nonagenarians who underwent colorectal resections demonstrated, by incorporating a physician’s input into the perioperative care, that the 30-day and 180-day mortality could be as low as 2.1 and 10.4%, respectively; patients in the study had a much lower (2%) incidence of pneumonia [16]. These encouraging results demonstrated the importance of multi-disciplinary care in the extreme elderly. In addition, patients undergoing laparoscopic surgery were shown to have better survival rates compared to those undergoing open surgery. However, this could be subjected to selection bias, as those undergoing open surgery may have more locally advanced disease and poorer cardiorespiratory reserve, which would prevent pneumoperitoneum. However, this echoed the finding of the above meta-analysis with lower incidence of respiratory complications and was in line with other studies that showed similar favorable short-term outcomes after laparoscopic colorectal surgery [10, 28]. Although such a finding was not confirmed in the current study, further research is warranted to evaluate the potential of laparoscopic surgery in improving surgical outcome in the extreme elderly.

This study was a retrospective analysis of patients from a single tertiary center with a relatively small number of patients. One major limitation was that the results were subjected to selection bias. The patients selected for surgery were regarded as having a reasonably good risk. A median follow-up period of 15.9 months was relatively short, but this was related to the limited lifespan of the patients who were senile to begin with. Moreover, only morbidity and mortality information were available, the quality of life and functional outcomes were not reported. Also, the study included a span of 20 years, in which some of the clinical practices had changed. For example, there was an increase in the use of laparoscopic approach since the year 2002, and the adoption of enhanced recovery after the surgery protocol in the year 2007.

## Conclusion

Advanced age alone should not preclude surgery in patients with colorectal cancer. A comprehensive risk profiling taking into account the co-morbidities, ASA, and CCI score is crucial. Nutritional status should be optimized, and measures should be taken to prevent postoperative pneumonia. A favorable outcome could be achieved in a selected group of nonagenarian patients.

## Data Availability

The dataset analyzed during the current study is available from the corresponding author on reasonable request.

## Abbreviations

ASA: American Society of Anesthesiologists
CCI: Charlson comorbidity index
ECOG: Eastern Cooperative Oncology Group
